# Prevalence of human papillomavirus and survival in oropharyngeal cancer other than tonsil or base of tongue cancer

**DOI:** 10.1002/cam4.2

**Published:** 2012-06-03

**Authors:** Linda Marklund, Anders Näsman, Torbjörn Ramqvist, Tina Dalianis, Eva Munck-Wikland, Lalle Hammarstedt

**Affiliations:** 1 Department of Clinical Science, Intervention and Technology, Department of Oto-Rhino-Laryngology, Head and Neck Surgery, Karolinska University Hospital, Karolinska InstitutetStockholm, Sweden; 2 Department of Oncology-Pathology, Karolinska InstitutetStockholm, Sweden

**Keywords:** HPV, HPV prevalence, oropharyngeal cancer, p16, survival

## Abstract

Today, most oropharyngeal squamous cell carcinoma (OSCC) is human papillomavirus (HPV) positive and HPV alone or in combination with p16 is reported to be a favorable prognostic factor for OSCC. Patients with tumors at other OSCC sites (OOSCC) are often included in the same treatment and study protocols as patients with tonsillar- and base of tongue SCC, even though the prevalence and clinical significance of HPV infection in OOSCC is unknown. Since tonsillar and base of tongue SSC cover roughly 90% of all OSCC, there is an obvious risk that there may be a misinterpretation of the results for OOSCC. Herein, we therefore study the prevalence of HPV and p16 and their impact on survival in OOSCC. A total of 69 patients were included in the study, and 61 were included in the survival analysis. HPV and p16 were present in only 17% (12/69) and 25% (17/69) of the OOSCC cases, respectively, while the majority 69% (48/69) was both HPV and p16 negative. Neither HPV nor p16 had predictive value for clinical outcome in OOSCC in this study. In conclusion, the prevalence of HPV and/or p16 is much lower in OOSCC compared to earlier reports including all OSCC, or tonsillar- and base of tongue cancer alone and HPV and p16 had no impact on clinical outcome in OSCC in this study. Our data highlight the diversity of head neck cancer sub-sites and the importance of taking OSCC sub-sites in consideration in future clinical trials and treatment.

## Introduction

Head and neck squamous cell carcinoma (HNSCC) is the sixth most common cancer worldwide [1]. Despite a decreasing incidence of HNSCC, in general, attributed to a decrease in the prevalence of smoking [2], the incidence of oropharyngeal SCC (OSCC) is rising, possibly due to an epidemic of human papillomavirus (HPV) infection [3–7]. HPV has for some time been suggested to be involved in the carcinogenesis of OSCC and the Agency for Research on Cancer (IARC) now recognizes HPV as a risk factor for OSCC. In several countries in the western world, the majority of OSCC cases are HPV positive, and in Stockholm, over 85% of the new cases of tonsillar- and base of tongue cancer are HPV positive [5–7]. In addition, HPV has been shown to be a favorable prognostic factor for patients with OSCC [8, 9] and more specifically for patients with tonsillar [1, 10–13] and base of tongue cancer [12, 14], leading to an increased interest in the predictive value of HPV.

Overexpression of the cyclin dependent kinase inhibitor 16, p16^INK4^ (p16) is strongly associated to HPV-positive OSCC [15–17], and can indicate biologically active HPV infection [16, 18]. p16 has therefore been proposed as a surrogate marker for HPV in clinical practice [19]. Even though the association of p16 to HPV is not absolute, it is suggested as a positive independent prognostic marker for tonsillar cancer and has also been shown to be a marker for increased sensitivity to radiotherapy [20]. It has thus been proposed to use p16 and HPV in combination to select patients with tumors sensitive to treatment [21].

Nevertheless, studies of OSCC do not always recognize the complexity of the region. Tonsillar and base of tongue SSC, both originating of the Waldener's ring, cover roughly 90% of all OSCC [22]. However, patients with tumors at other OSCC (OOSCC) sites are often included in the same treatment and study protocols as patients with tonsillar- and base of tongue SCC, even though the prevalence and clinical significance of HPV infection in OOSCC is unknown. The consequence may be a misinterpretation of the results.

For this purpose, we herein study the prevalence of HPV and p16 in the OOSCC sites separately, as well as their impact on patient survival.

## Patients, Materials, and Methods

### Patient data

All patients diagnosed between 2000 and 2008, in the County of Stockholm, Sweden, with OSCC outside the tonsils and base of tongue (OOSCC), where pretreatment biopsies were available were included in this study. The International Classification of Diseases (ICD) system 10, where the oropharynx is subdivided into the anterior wall, that is, the base of tongue and vallecula; the lateral wall, that is, the tonsillar fossa; the posterior wall; the superior wall; and the soft palate, was used for diagnosis. OSCC C10.0–C10.9 and C50.1–C50.8 were included in the analysis. Sub-site C50.8 was analyzed to assess tumors from the soft palate; however, one patient was excluded due to the origin of the tumor in the hard palate. All patients' charts were analyzed, and age, gender, TNM-stage, treatment, and survival were recorded. Treatment was categorized as surgery, radiotherapy, including both external and interstitial (brachytherapy) radiation sources, with doses up to 68 Gray, or chemoradiotherapy. Patients were evaluated for disease progression every 3 months the first 3 years, then every 6 months for a total of 5 years. All patients were evaluated for a minimum of 3 years, and the follow-up period ranged from 1 to 122 months. For the survival analysis, age was categorized into two groups, either under or over the age of 67 (mean age for the whole group). Patients categorized as Stages I or II at time of diagnosis were pooled into one group and compared to patients categorized as Stages III and IV. The study was conducted according to ethical permissions 2005/431-31/4 and 2005/1330-32 and 2009/1278-31/4 from the Ethical Committee at Karolinska Institutet, Stockholm, Sweden.

### Detection of HPV-DNA

HPV-DNA was detected as previously [5, 6, 23]. Briefly, DNA was extracted from 30 μm paraffin-embedded tumor biopsies and HPV-DNA was analyzed using PCR using general primer pairs GP5+/6+. The samples negative for GP5+/6+ were further tested using CPI/IIG primers to avoid false-negative results. Type-specific primers for HPV-16 and sequencing were also used. In addition, presence of amplifiable DNA was tested by the human housekeeping gene S14 PCR.

### Immunohistochemical analysis of p16^INK4a^

Immunohistochemistry (IHC) was performed with the p16^INK4a^ primary monoclonal mouse anti-human p16^INK4a^ antibody (dilution 1:100; clone JC8; Santa Cruz Biotechnology, Inc., Santa Cruz, California). In brief, 4-μm sections of formalin-fixed and paraffin-embedded tissue were deparaffinized in xylene and re-hydrated in graded alcohol. Epitope retrieval in citrate buffer (pH 6) in micro-oven for 20 min and cooled on bench followed by peroxidase blocking (3% H_2_O_2_) for 10 min. Slides were then blocked with BSA followed by incubation with the primary monoclonal antibody over night (8°C). Secondary biotynilated anti-mouse antibody was added and was followed by a standard avidin-biotin-complex-PO technique using the VECTASTATIN® Elite® ABC kit (Vector Laboratories, Inc., Burlingame, California). Slides were developed in DAB and counterstained in hematoxylin. p16 staining was graded on a four-tier scale (0: 0%; 1: 1%–25%; 2: 26%–74%; 3: 75%–100%). Samples with >75% p16-positive tumor cells were considered as p16 positive and notably for all tumors either >90% or <10% of the tumor cells were p16 positive.

### Statistical analyses

The Fischer's exact test was used to compare differences in tumor stage, gender, and treatment between HPV-positive, p16-positive, and negative samples. Kruskal–Wallis test was used to compare age in the different HPV and p16 groups. Disease-free survival was calculated from the end of treatment until any recurrence. Deaths from other causes or patients lost to follow-up were censored. Overall survival was calculated from date of diagnosis to death of any cause. The HPV-positive cases were compared to the HPV-negative cases and analyzed using the Kaplan–Meier method, and survival curves were compared by use of the log-rank test in the univariate analysis. A similar analysis was made comparing HPV-positive and p16-positive cases to the rest. In the multivariate analysis, a Cox proportional hazards model was used to adjust for all covariates in the univariate analysis. STATA was used for the survival analysis, whereas Fischer's exact test and Kruskal–Wallis test was calculated using VassarStat website (http://faculty.vassar.edu/lowry/VassarStats.html).

## Results

### Presence of HPV and p16 in oropharynx squamous cell cancer outside the tonsil and tongue base

Tumors from 69 patients (49 male and 20 female patients) of the 75 patients with OOSCC, with correct diagnosis and available pretreatment biopsies were included in the HPV and p16 analysis. Patients and tumor characteristics are summarized in Table 1. The age of the patients at the time of diagnosis ranged from 45 to 95 years, with a mean age of 67 years. Twenty-six patients had a tumor originating from the soft palate (C05.1–C05.8) and 43 patients had tumors originating from the oropharynx (C10).

**Table 1:** Characteristics of patients and tumor samples between 2000 and 2008

			HPV+^2^		HPV−2		
					
Patient and tumor characteristics^1^	All patients	HPV analyzed tumors^2^	p16+^2^	p16−2	p16+^2^	p16−2	*P*-value^4^
Total number	75	69	8	4	9	48	
Gender							
Male	52	49	5	3	9	32	0.172
Female	23	20	3	1	0	16	
TNM classification^3^							
T1	9	9	2	1	1	5	0.55
T2	25	21	2	2	2	15	
T3	25	24	3	1	5	15	
T4	16	15	1	0	1	13	
N0	29	27	2	2	7	16	0.07
N+	44	41	6	2	2	31	
Nx	2	1	0	0	0	1	
M0	64	62	8	4	8	42	0.99
M1	2	2	0	0	0	2	
Mx	9	5	0	0	1	4	
Stage^3^							
I	3	3	0	1	0	2	0.19
II	11	9	0	1	1	7	
III	25	23	4	1	5	13	
IV	36	34	4	1	3	26	
Mean age^4^	67 (47–95)	66 (45–95)	70 (59–84)	59 (47–70)	63 (45–85)	68 (49–95)	0.94
Treatment							
RT	49	45	7	2	5	31	0.57
CRT	5	5	0	1	1	3	
Surgery	6	6	1	0	2	3	
Surgery+RT	4	4	0	0	1	3	
Palliative/NT	11	9	0	1	0	8	

1 At time of diagnosis.

2 Presence of HPV-DNA and expression of p16 in tumors analyzed using PCR and IHC, respectively.

3 TNM classification and stage of cancer according to International Union Against Cancer, UICC, 2002.

4 *P*-value calculated by Fischer's exact test. For T-stage, T1 + T2 was compared with T3 + T4. For stage, stage I + II was compared with stage III + IV. Kruskal–Wallis test was used for calculation of *P*-value in mean age group.

Of all tumors, only 17% (12/69) were HPV positive (10 HPV16, 1 HPV18, and 1 HPV33), and only 25% (17/69) were p16-positive (Table 1). Of the HPV-positive tumors, 67% (8/12) were p16 positive, while 16% (9/57) of the HPV-negative tumors were p16 positive. The majority of the tumors 69% (48/69) were both HPV and p16 negative, while only a minority, 12% (8/69) of the tumors, was both HPV and p16 positive. Noteworthy, only 47% (8/17) of the p16-positive tumors were also HPV positive.

There were no significant differences for the presence or absence of HPV or p16 between the different sub-sites (Table 2). There were also no significant differences regarding mean age, gender, or stage between patients with HPV-positive and HPV-negative tumors.

**Table 2:** HPV and p16 prevalence in different sub-sites^1^

		HPV+^2^		HPV−2	
			
Sub-site	All patients (*n* = 69)	p16+^2^ (*n* = 8)	p16−2 (*n* = 4)	p16+^2^ (*n* = 9)	p16−2 (*n* = 48)
C051–C058	26	2 (8)	3 (12)	5 (19)	16 (62)
C101–C109	43	6 (14)	1 (2)	4 (9)	32 (74)

Values are expressed as *n* (%).

1 *P* = 0.212 Fischer's exact test.

2 Presence of HPV-DNA and expression of p16 in tumors analyzed using PCR and IHC, respectively.

### Survival analysis in patients with OOSCC in correlation to HPV and p16

For the survival analysis, 61 OOSCC patients were available of the original 75 patients, after excluding those treated with palliative intent (*n* = 11) and those where biopsies were not available for HPV analysis (*n* = 6). Of the six patients, where biopsies (four with C05 and two with C10) could not be obtained, three were treated with palliative intent. All patients with HPV-positive and p16-positive tumors were included in the analysis. The mean age in the group not available for HPV analysis was 64 years. Further characteristics of the patients and their tumors are listed in Table 1.

The mean follow-up time was 34 months (range 1–122). In all, 49 patients died during follow-up, seven patients with HPV-positive cancer, and 42 with HPV-negative cancer. However, of these, three patients with HPV-positive cancer and five patients with HPV-negative cancer died free of disease. Four patients with HPV-positive tumors (33%) and 27 patients with HPV-negative tumors (55%) had recurrence.

**Figure 1:**
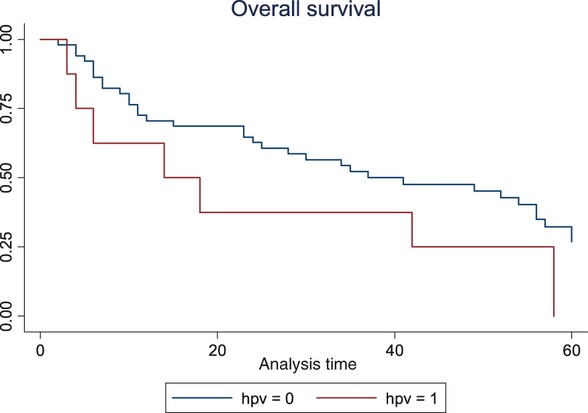
Overall survival illustrated with Kaplan–Meier curves comparing patients with HPV-positive and HPV-negative tumors. *P*-value 0.622 (log-rank test).

**Figure 2:**
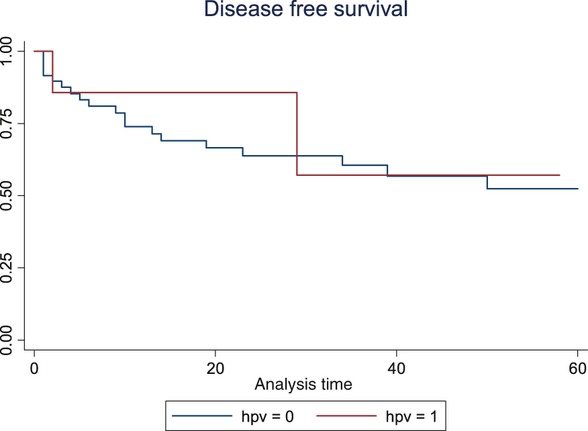
Disease-free survival illustrated with Kaplan–Meier comparing patients with HPV-positive and HPV-negative tumors. *P*-value 0.618 (log-rank test).

There was no prognostic significance for HPV (Table 3) or HPV in combination with p16 (data not shown), in the overall or the disease-free survival analysis for OOSCC (Figs 1 and 2).

**Table 3:** Univariate and multivariate models for overall and disease free survival

	Univariate			Multivariate		
		
Variable	Hazard ratio	*P*	95% CI	Hazard ratio	*P*	95% CI
Overall survival						
Age (years)						
<67	1.00		Reference	1.00		Reference
>67	1.61	0.125	0.88–2.98	1.44	0.297	0.49–2.10
Gender						
Female	1.0		Reference	1.0		Reference
Male	1.08	0.827	0.54–2.15	1.08	0.841	0.53–2.20
Stage						
I–II	1.0		Reference	1.0		Reference
III	1.75	0.237	0.69–4.40	1.48	0.425	0.56–3.88
IV	1.98	0.145	0.79–4.97	1.96	0.184	0.73–5.30
Treatment						
Surgery	1.0		Reference	1.0		Reference
RT	0.52	0.176	0.20–1.35	0.54	0.229	0.20–1.47
ChemoRT	0.44	0.258	0.10–1.84	0.50	0.372	0.11–2.30
RT+surgery	0.73	0.643	0.19–2.74	0.76	0.697	0.19–3.04
HPV−	1.0					
HPV+	1.21	0.625	0.56–2.64	1.38	0.442	0.61–3.16
Disease-free survival						
Age (years)						
<67	1.00		Reference	1.00		Reference
>67	1.24	0.622	0.53–2.88	1.65	0.308	0.63–4.31
Gender						
Female	1.0		Reference	1.0		Reference
Male	0.83	0.695	0.34–2.06	0.73	0.521	0.27–1.92
Stage						
I–II	1.0		Reference	1.0		Reference
III	6.34	0.078	0.81–49.57	6.61	0.076	0.82–53.28
IV	6.72	0.069	0.87–52.22	7.05	0.070	0.85–58.11
Treatment						
Surgery	1.0		Reference	1.0		Reference
RT	1.91	0.531	0.25–14.57	1.72	0.607	0.22–13.80
ChemoRT	3.53	0.275	0.37–34.08	3.26	0.329	0.30–34.95
RT+surgery	3.30	0.301	0.34–31.83	2.57	0.427	0.25–26.35
HPV−	1.0					
HPV+	0.74	0.623	0.22–2.49	0.82	0.759	0.23–2.92

## Discussion

In this study, we analyzed OSCC outside the tonsillar fossa and base of tongue (OOSCC) for presence of HPV-DNA and expression of p16 and only found a minority (16%) to be HPV positive and 25% to be p16 positive. In addition, there was no complete correlation between HPV status and p16. Furthermore, in contrast to a clear positive impact of HPV and p16 on clinical outcome in tonsillar and base of tongue cancer, this was not the case for OOSCC.

The low prevalence of HPV in OOSCC obtained in this study (16%) could be due to the fact that the epithelial tissue of OOSCC differs from that in tonsillar and base of tongue cancer. In addition, an even lower prevalence of HPV was observed when assessing tumors positive for both HPV and p16 (12%). Our data were well within the range of HPV prevalence in OOSCC reported earlier (0%–60%) [24–28] however, some of these previous studies include cohorts with fewer patients. The HPV prevalence was 21% in a study by Licitra et al. [24] including 24 OOSCC patients, and 17% in a study by Hong et al. [25] including 35 patients with OOSCC. Furthermore, in a study by Lindel et al. [29], including 59 non-tonsillar OSCC patients, the HPV prevalence was 14%, but the authors did not specify whether patients with base of tongue cancer were included in that group or not.

Notably, in this study, in the survival analysis including 61 patients, it was not obvious that the presence of HPV or p16 showed a clear-cut benefit for prognosis in OOSCC. This is in contrast to several previous studies that have shown that prognosis for OSCC patients with HPV-positive cancer is clearly better than for those with HPV-negative cancer independent of nodal status, age, stage, tumor differentiation, or gender [1, 8, 9, 11, 21, 30]. More specifically, some reports show an 80%–95% 2–3 year overall survival rate for patients with HPV-positive OSCC as compared to 57%–62% for those with HPV-negative OSCC [8, 9, 14]. The improved survival for patients with HPV-positive OSCC is regardless of treatment strategy, and there is a debate today, whether intensified therapy is unnecessarily aggressive in this group of patients, since they show a superior survival regardless of treatment strategies. However, many of these studies included OSCC as a group, and the contribution of OOSCC is either small or not specified, and since OOSCC only covers roughly 10% of OSCC, it is both possible and likely that a potential difference survival is not made visible. This fact stresses the need for sub-site specification when analyzing head and neck cancer. The overall survival in this group of patients is low, even with a low T-stage. This fact might indicate a need for more aggressive treatment in this group of patients (P. Attner, J. Du, L. Hammarstedt, J. Lindholm, E. Munck-Wikland, T. Ramqvist, T. Dalianis, and L. Marklund, pers. comm., 2011).

Also, in contrast to earlier data showing a very high correlation between presence of p16 and HPV in OSCC [20], we found that only 47% of the p16-positive tumors were also HPV positive, and thus our study showed a markedly lower association. To our knowledge, all previous studies were either performed on OSCC as a group or including only tonsillar cancer [15, 16, 20, 31], which explains the difference in correlation between the markers. On the basis of our results in OOSCC, we would not recommend the use of p16 as a surrogate marker for HPV status for OOSCC patients.

There are some limitations in our study and first of all this is a retrospective study with its potential bias involved. However, since OOSCC is a rare disease, it is difficult to study prospectively, and to our knowledge, this is the largest presented data on non-tonsillar, non-base of tongue OSCC. Nevertheless, there was still a limitation in the study sample size, which may explain the lack of significance in the survival analysis in the age and stage group. However, there was a clear trend toward a worse outcome for patients with a higher stage and age. Moreover, the quality of the data on smoking status and comorbidity was poor and inconsistent; thus, we were not able to adjust for these factors. Even though there are limitations in this analysis, there is still a marked difference in survival when compared to similar studies with similar sample sizes [14].

The fact that the prevalence of HPV was lower than that in tonsillar and base of tongue cancer was not so controversial, since it was similar to that in other reports [24]. Still, as mentioned above, there was no difference in survival detected for patients with HPV-positive or HPV-negative OOSCC, irrespective of whether HPV and p16 were analyzed solely or together. This was also the case for both overall survival and disease-free survival. This could of course be due to the small sample size and the low number of patients with HPV-positive tumor; hence, the results must be interpreted with caution. Another possibility is that the biological activity and function of HPV differ in OOSCC as compared to that in tonsillar and base of tongue cancer, where the last two comprise the Waldener's ring. Irrespectively, it is notable that the survival trend for OOSCC patients differs from the survival trends observed for both tonsillar- and base of tongue cancer, where a clear difference and survival benefit for patients with HPV-positive cancer is seen in previous studies [1, 10–14]. Due to sample size constraints the conclusions from this analysis can only be indicative but it is marking a possible difference which should be addressed in future studies on oropharyngeal cancers.

In summary, the prevalence of HPV and p16 is relatively low in OOSCC, and HPV and p16 could not be used here independently or in combination to show an impact on survival. In this limited cohort the correlation between HPV status and/or p16 in the tumors was not as strong as shown in previous studies including all OSCC or tonsillar SCC [15, 16, 20, 31–33]. In conclusion, our data implicate the significance of analyzing different OSCC sub-sites separately, especially when considering the influence of HPV and p16 on clinical outcome, and when planning future studies and treatment protocols for the different sub-sites.
